# Early Trajectories of Resting-State EEG power in autistic children: a longitudinal study across language profiles

**DOI:** 10.1038/s41398-026-04132-0

**Published:** 2026-05-25

**Authors:** Kenza Latrèche, Michel Godel, Ana Flò, Fiona Journal, Valentina Borghesani, Marie Schaer

**Affiliations:** 1 Autism Brain and Behavior Lab, Faculty of Medicine, University of Geneva, Geneva, Switzerland; 2 Division of Adult Psychiatry, Department of Psychiatry, University Hospitals of Geneva, Geneva, Switzerland; 3 Department of Psychiatry, University School of Medicine, Geneva, Switzerland; 4 Cognitive Neuroimaging Unit, Université Paris Saclay, NeuroSpin center, Gif-sur-Yvette, France; 5 Department of Developmental Psychology and Socialisation and Department of Neuroscience, University of Padova, Padova, Italy; 6 Neurobiology of Concepts Expression Laboratory, Faculty of Psychology and Educational Sciences, University of Geneva, Geneva, Switzerland

**Keywords:** Diagnostic markers, Neuroscience

## Abstract

Language development in autism spectrum disorder (ASD) is heterogeneous, ranging from subtle differences to significant delays. In previous work, we identified three autistic language profiles in early childhood: Language Unimpaired (LU), Language Impaired (LI), and Minimally-Verbal (MV). While these profiles show distinct vocabulary, grammar, and pragmatic development, understanding their underlying neural correlates is essential to predict outcomes and develop targeted interventions. Here, we examined whole-brain resting-state EEG power across five canonical frequency bands in a longitudinal sample comprising 66 typically developing (TD) children and 122 autistic children (ages 1.6–6.0 years), yielding 358 time points. Within the ASD group, 61 children belonged to the LU profile, 44 children to LI, and 17 children to MV. Compared to TD peers, autistic children showed increased power in low-frequency (delta, theta) and high-frequency bands (beta, gamma). Gamma power varied by autistic language profile, with the highest levels in MV children. Moreover, gamma power within ASD followed a quadratic trajectory in relation to word combination acquisition, peaking around the time of acquisition and decreasing afterward. This pattern suggests a dynamic, compensatory mechanism supporting the transition to phrase speech, which is a critical milestone toward functional speech that may predict language outcomes in ASD.

## Introduction

Autism Spectrum Disorder (ASD) is a prevalent neurodevelopmental condition characterized by difficulties in social communication and interactions, as well as repetitive and restrictive behaviors or interests [1]. While outcomes across the spectrum vary widely, early language milestones (e.g., age of first spoken words), are one of the strongest predictors of long-term development [2–4]. However, language development in autism ranges from significant difficulties to subtle differences [5, 6]. In previous work, we aimed to capture language heterogeneity among 215 autistic children (aged 1.5–5.7 years old) [7]. Using a data-driven approach, we identified three distinct clusters that corresponded to the autistic language profiles described in the 11th edition of the International Classification of Diseases, namely Minimally-Verbal (MV), Language Impaired (LI), and Language Unimpaired (LU) [8, 9]. In our previous study, MV children exhibited poor vocabulary and limited use of word combinations [7]. The LI group showed delayed vocabulary development and reduced use of word combinations, while the LU group demonstrated vocabulary size and word combinations comparable to typically developing (TD) peers [7].

Understanding the neural correlates underlying these distinct language trajectories is crucial for predicting outcomes and developing targeted interventions, particularly for children with greater language difficulties [10–12]. Electroencephalography (EEG) has emerged as a powerful tool in research in autistic children, given its high temporal resolution and non-invasiveness [13]. However, EEG data collection can be challenging in young autistic children due to anxiety about unfamiliar experiences and/or tactile defensiveness [14, 15]. Habituation strategies (i.e., preparatory procedures aimed at familiarizing children with the EEG environment) have been shown to improve compliance, including in MV children [14, 16].

Resting-state EEG (RS-EEG) paradigms are particularly valuable in this context, as they require no engagement in a cognitive task [13]. RS-EEG activity is frequently examined with spectral analysis, in which brain oscillations are decomposed and power is quantified across five canonical frequency bands: delta (2–4 Hz), theta (4–6 Hz), alpha (6–13 Hz), beta (13–30 Hz), and gamma (30–50 Hz) [13, 17, 18]. Prior evidence suggested a U-shaped profile in ASD, with increased power for low frequencies (delta, theta) and high frequencies (beta, gamma) and decreased power for middle frequencies (alpha) compared to TD [13, 18, 19]. Nevertheless, reliable RS-EEG biomarkers of ASD have yet to be identified, due to limitations in previous studies including small sample sizes (on average 30 autistic and 35 TD participants [18]), predominantly cross-sectional, and analyses limited to specific electrodes and/or frequency bands [17].

Some previous studies examined the relationship between RS-EEG and language development in infants at high- and low-likelihood (HL; LL) for autism [20, 21]. In a cross-sectional study on 70 HL infants aged 12–23 months, Cohenour and colleagues reported positive correlations between alpha power and receptive and expressive language skills, suggesting that alpha oscillations support language production and comprehension [20]. In a longitudinal study in 72 HL and 58 LL infants, increased 6-month frontal gamma power (FGP) was significantly associated with higher 24-month language functioning in HL infants without ASD (HL-NoASD), but with lower 24-month language functioning in HL infants later diagnosed with ASD (HL-ASD) [21]. As gamma oscillations reflect the brain’s balance between excitatory/inhibitory (E/I) neurotransmitters, reduced FGP in HL-ASD could represent successful compensation for underlying neurotransmitter imbalances. In contrast, reduced FGP in HL-NoASD could indicate delayed neural maturation and language abilities [21]. To more accurately assess the brain’s E/I balance, Wilkinson and colleagues analyzed aperiodic power (i.e., non-oscillatory, broad band neuronal spiking activity) in a subsequent longitudinal study of 3- and 12-month-old HL and LL infants [22]. They found greater increases in aperiodic power in HL-ASD infants, along with reduced language development at 18 months, suggesting that early E/I imbalance may underlie later language difficulties in HL-ASD infants. Furthermore, a cross-sectional study on preschoolers (48 ASD, 58 TD) showed decreased FGP associated with greater expressive language skills in autistic children [23], supporting that reduced gamma power may reflect a compensation mechanism for language development [21, 23].

While previous research has reported some associations between RS-EEG power and language development, it remains difficult to draw a comprehensive understanding of the relationships between EEG frequency bands and language outcomes in autism. This gap can be attributed to three main limitations in existing literature: (1) a predominant focus on isolated frequency bands rather than the full spectrum of neural oscillations; (2) samples restricted to narrow age ranges; and (3) a lack of longitudinal study designs capable of tracking trajectories of both language development and RS-EEG power. Longitudinal investigation is particularly important as RS-EEG power trajectories might change non-linearly during childhood, alongside language development [17, 21]. To our knowledge, no prior study has longitudinally examined the five canonical frequency bands of RS-EEG power in autistic children stratified by language profile. To address these limitations, the present study conducts a longitudinal investigation of whole-brain EEG power in the five canonical frequency bands in three previously validated autistic language profiles (LU, LI, and MV) [7]. The longitudinal sample yields 358 time points and includes 122 autistic (61 LU, 44 LI, 17 MV) and 66 TD children, aged 1.56–6.01 years old. Our study has three aims, (1) to explore longitudinal trajectories of each frequency band across the TD and ASD groups, and within LU, LI, and MV; (2) to examine the trajectory of gamma power in relation to word combination acquisition within the ASD sample; (3) to classify TD and the three autistic profiles based on fine-grained brain activity features, using a supervised machine learning algorithm. We hypothesized that children with ASD would demonstrate increased power in low (delta, theta) and high frequency bands (beta, gamma) and decreased power in middle frequencies (alpha), in line with the proposed U-shaped profile [18]. We expected the LU, LI, and MV subgroups to differ mainly in the gamma band, reflecting previously reported associations between language skills and higher frequencies in autistic children and HL infants [21, 23]. Assuming that decreased gamma power may reflect a compensatory mechanism in ASD, we hypothesized that MV would exhibit the greatest gamma power, followed by LI, and LU. Considering that production of word combinations greatly differed across each language profile [7], we evaluated gamma power trajectory jointly with this milestone.

## Material and methods

### Participants

Our sample comes from the Geneva Autism Cohort, which is an ongoing open longitudinal cohort that follows autistic and TD preschoolers [7]. In this cohort, we include children who received a diagnosis of ASD before age 5, and their TD peers, and we follow them every 6 months for two years. Each visit comprises several longitudinal assessments, from behavioral measures to neuroimaging. Autistic participants are recruited through local clinical centers and parent associations. A licensed child psychiatrist (MS) confirmed the diagnosis of all autistic participants based on the *Diagnostic and Statistical Manual of Mental Disorders -* 5^th^ ed. [1] and gold-standard tools (Autism Diagnostic Observation Schedule, Second Edition [24]; Autism Diagnostic Interview-Revised [25]). Inclusion criteria for TD participants included full-term birth, no ASD diagnosis in first-degree relatives, and no neurological or somatic concerns. TD participants completed the ADOS [24] to exclude ASD symptoms and a developmental assessment (either the Mullen Scales of Early Learning, MSEL [26] or the Psychoeducational Profile – third edition, PEP-3 [27]) to rule out significant developmental delays (i.e., a developmental quotient below 70 in any domain). Written informed consent forms were signed and provided by the participants’ caregivers. The Ethics Committee of the University of Geneva approved the research protocol.

### Determination of language profiles

Language profiles, i.e., Language Unimpaired (LU), Language Impaired (LI), and Minimally-Verbal (MV), were established in our previous study using the same longitudinal cohort [7]. Thus, the present study does not redefine these profiles but applies the previously published classification to a subset of participants. Specifically, we included all autistic and TD participants from our previous work [7] who had at least one RS-EEG recording.

Briefly, in our previous study [7], we identified a stable verbal outcome age by examining longitudinal changes in expressive and receptive language developmental quotients (DQs; calculated as expressive and receptive developmental age scores divided by chronological age × 100; see Measures section below for details). Language DQs stabilized by 3.75 years of age, allowing verbal outcome to be defined using only assessments obtained after this age. A data-driven TwoStep clustering algorithm was then applied to expressive and receptive language DQs at the outcome age of 4.4 years (i.e., the mean age of the ASD sample after removing all time points collected before 3.75 years), yielding three clusters corresponding to MV, LI, and LU. Full methodological details are provided in our previous work [7].

The final sample comprised 188 participants (122 autistic, 66 TD, 1.56–6.01 years) contributing 358 EEG recordings. Among autistic participants, subgroup membership was as follows: LU (61 participants, 127 time points, 1.74–5.99 years, 6.6% females), LI (44 participants, 71 time points, 1.93–5.82 years old, 11.4% females), and MV (17 participants, 30 time points, 1.80–6.01 years old, 35.3% females). Sample characteristics are reported in Table 1.

**Table 1:** Sample characteristics of the TD and LU, LI, and MV samples.

Measure (mean (*SD*))	TD	ASD	ASD-LU	ASD-LI	ASD-MV
Number of participants	66	122	61	44	17
Number of time points	130	228	127	71	30
Time points per participants	2.0 (*0.8*)^‡^	1.9 (*0.8*)	2.1 (*0.8*)	1.6 (*0.7*)	1.8 (*0.8*)
Mean age at first available TP (years)	3.3 (*1.1*)^‡^	3.5 (*1.0*)	3.4 (*0.9*)	3.8 (*1.1*)	3.2 (*1.3*)
Age range (years)	1.6–5.8	1.7–6.0	1.7–6.0	1.9–5.8	1.8–6.0
Female biological sex	29 (43.9%)^‡^	15 (12.3%)^†^	4 (6.6%)	5 (11.4%)	6 (35.3%)
Multilingual environment [*N* = 188]	24 (36.4%)	57 (46.7%)	27 (44.3%)	18 (40.9%)	12 (70.6%)
Parental education [*N* = 188] (% college degree completed)	62 (93.9%)^‡^	65 (53.7%)^†^	28 (45.9%)	24 (54.5%)	9 (52.7%)
Mean ADOS CSS Total at first available TP [*N* = 188]	1.1 (*0.3*)^‡^	7.3 (*1.8*)^†^	6.7 (*1.6*)	7.5 (*2.0*)	8.7 (*1.4*)
Mean ADOS CSS SA at first available TP[*N* = 188]	1.1 (*0.3*)^‡^	6.3 (*2.1*)^†^	5.8 (*1.9*)	6.4 (2*.4*)	7.6 (*1.7*)
Mean ADOS CSS RRB at first available TP [*N* = 188]	2.6 (*2.1*)^‡^	8.6 (*1.6*)^†^	8.4 (*1.8*)	8.7 (*1.5*)	9.3 (*1.1*)
Mean Total DQ [TP = 356]	114.8 (*12.5*)^‡^	79.14 (*25.6*)^†^	94.5 (*15.7*)	65.7 (*13.4*)	36.6 (*11.5*)
Mean EL DQ [TP = 351]	107.8 (*18.2*)^‡^	66.6 (*27.3*)^†^	84.3 (*18.4*)	53.8 (*15.0*)	23.8 (*9.9*)
Mean RL DQ [TP = 351]	118.3 (*14.3*)^‡^	72.9 (*32.1*)^†^	93.6 (*20.3*)	58.5 (*21.0*)	21.6 (*9.3*)
Mean VR DQ [TP = 351]	123.3 (*20.5*)^‡^	88.3 (*27.5*)^†^	104.4 (*20.3*)	77.1 (*17.0*)	48.6 (*17.8*)
Mean FM DQ [TP = 351]	107.9 (*13.8*)^‡^	81.1 (*22.2*)^†^	94.0 (*17.0*)	73.4 (*15.4*)	52.2 (*16.8*)
DLPF Total Vocabulary Score [TP = 322]	1104.0 (*412.3*)^‡^	641.9 (*503.2*)^†^	852.6 (*478.3*)	454.7 (*355.7*)	24.4 (*69.8*)
DLPF Total Grammar Score [TP = 323]	79.6 (*29.9*)^‡^	42.1 (*36.9*)^†^	57.7 (*36.6*)	26.7 (*22.8*)	0.79 (*2.6*)
DLPF Total Pragmatics Score [TP = 314]	45.2 (*6.3*)^‡^	30.5 (*16.8*)^†^	38.4 (*12.2*)	26.8 (*13.4*)	2.4 (*4.3*)

Probability values refer to Kruskal–Wallis, Mann-Whitney, or Pearson chi-square tests, as appropriate.

^†^*p* < .05 vs. TD; ^‡^*p* < .05 across TD, LU, LI, MV.

*ADOS* autism diagnostic observation schedule, *ASD* autism spectrum disorder, *ASD-LU* autism spectrum disorder-language unimpaired, *ASD-LI* autism spectrum disorder-language impaired, *ASD-MV* autism spectrum disorder-minimally-verbal, *CSS* calibrated severity score, *DLPF* développement du langage productif en français, *DQ* developmental quotient, *EL* expressive language, *FM* fine motor, *RL* receptive language, *RRB* restricted and repetitive behaviors, *SA* social affect, *TD* typical development, *TP* time point, *VR* visual reception.

We used G*Power to conduct a power analysis of our sample. With our TD group (*N* = 66) and ASD group (*N* = 122), the study is well-powered to detect both large (Cohen’s d = 0.8) and medium (Cohen’s d = 0.5) effect sizes. Power was nearly 100% for a large effect and 90.6% for a medium effect, indicating sufficient sample sizes to detect meaningful differences across groups. Detecting a small effect (Cohen’s d = 0.2) would require 394 participants per group to achieve a power of 80%. Of note, this analysis does not account for the repeated measures within participants. As no clear consensus exists on power analysis for unbalanced, nested longitudinal designs, we report cross-sectional power estimates for informational purposes, acknowledging the limitations of this approach.

### Measures

To characterize our longitudinal cohort, behavioral and developmental measures were administered to complement the neurophysiological data.

#### Behavioral measures

We administered the ADOS [24] to confirm ASD in autistic participants and to exclude ASD symptoms in TD participants. The ADOS is a semi-structured assessment quantifying symptoms in social affect and restricted and repetitive behaviors or interests. The ADOS includes five modules, administered depending on the child’s age and language level. Modules were compared using calibrated total severity score [28, 29]. The ADOS was administered and coded by trained examiners.

Developmental level was measured using the MSEL [26], assessing fine motor (FM), visual reception (VR), receptive language (RL), and expressive language (EL) in children aged 0–68 months. We computed developmental quotients (DQs) by dividing the developmental age by the chronological age and multiplying by 100 [7, 30, 31]. DQs were selected over standard scores to reduce floor-effects in lower-performing participants. As the MSEL was added later in our protocol, 23 participants (32 time points) were assessed with the PEP-3 [27] or with the WPPSI-IV [32]. For the PEP-3, designed for children aged 2–7 years, we computed DQs for domains. The PEP-3 and MSEL showed excellent consistency for both expressive (Cronbach’s alpha = 0.899) and receptive language (Cronbach’s alpha = 0.913) [7]. Five children (5 time points) completed the WPPSI-IV, where only the full-scale IQ was used. Two children (1 ASD, 1 TD) had no developmental data at the EEG timepoint: one was too young, the other had behavioral difficulties invalidating the PEP-3.

#### Parent-reported expressive language

Expressive language skills were evaluated with the *Développement du langage de production en français* (DLPF) [33] parent-reported questionnaire. The DLPF assesses vocabulary, grammar, and pragmatic skills. Three total domain scores were computed [34]. The DLPF assesses children aged 18–42 months, as scores plateaued when approaching 42 months in TD [33]. Here, we administered the DLPF up to 6 years of age given the frequent expressive language delay in ASD [35].

We also examined word combination acquisition, which is a significant milestone toward functional speech [36]. We defined word combination as the use of at least two words to communicate (e.g., *Open door*, *More milk*) [33, 36]. Word combination acquisition was operationalized as a binary variable (acquired vs. not acquired), reflecting the emergence of this milestone.

The age of word combination acquisition was determined using the standardized assessment available at each time point in our longitudinal protocol, including the parent-report questionnaire *Développement du langage de production en français* (DLPF; Sentences and Grammar section, item B), the Autism Diagnostic Interview–Revised (ADI-R; item 10), the Mullen Scales of Early Learning (MSEL; Expressive Language scale, item 17), or the Autism Diagnostic Observation Schedule, Second Edition (ADOS; item A1). These instruments differ in their operational definitions of word combinations, particularly with respect to spontaneous speech and echolalia. Accordingly, word combination acquisition was defined consistently across time points as the presence of utterances containing at least two words, regardless of whether the utterance was echoed or spontaneous, based on the available measure.

#### RS-EEG data collection, preprocessing, and analyses

RS-EEG recordings were acquired with a sampling rate of 1000 Hz using 128-channel Hydrocel Geodesic Sensor Nets and Net Station acquisition software (v 5.4.1.2) (Magstim EGI, Inc. Eugene, OR). As EEG data collection can be challenging, we used several habituation strategies prior to the child visit to help children and their parents familiarize themselves with the EEG (e.g., pictograms, a short story illustrating the EEG room and net, a video demonstrating the setup and procedure, and a mock EEG net). Moreover, to minimize movement artifacts and facilitate compliance, we adapted the RS-EEG paradigm: children watched an age-appropriate cartoon of their choice with sound, sitting ~60 cm from the screen, independently or on a caregiver’s lap. They were encouraged to sit still and quiet. EEGs were recorded for 5 min and extended by 1–2 min if children were initially agitated or talking. We excluded 16 recordings under 300 s (5 from TD, 2 from LU, 8 from LI, 1 from MV). For longer recordings, the last 300 s were selected to retain the most stable segments.

As the present study is based on the dataset of our prior work, we selected all children who were administered at least one RS-EEG recording. From our previous sample sizes [7], we included 77.6% of TD participants (66/85) and 56.7% of participants with ASD (122/215), which is consistent with previous reports of EEG compliance difficulties in ASD (χ²(1) = 11.38, *p* < 0.001) [14, 15]. Regarding the language profiles, we included 70.9% of LU participants, 51.8% of LI, and 38.6% MV participants (χ²(2) = 13.79, *p* = 0.001), indicating greater challenges with limited language [16].

#### RS-EEG preprocessing and analyses

Preprocessing was conducted using the Automated Pipeline for Infants Continuous EEG (APICE) [37], which is based on EEGLAB [38] and is specialized for young children. The data were first low-pass filtered at 100 Hz and high-pass filtered at 1 Hz, and a notch filter was applied to remove line noise (50 Hz). The pop_eegfiltnew function from EEGLAB was used for filtering with the default parameters. Then, APICE was applied. APICE detects non-functional electrodes and motion artifacts based on signal properties, primarily fast signal changes, and repairs the data, when possible, to maximize data recovery. Brief artifacts (shorter than 100 ms) were corrected using PCA, while longer artifacts affecting only a subset of the channels (less than 30%) were corrected using spatial spline interpolation. Channels identified as non-functional throughout the recording were also interpolated using spatial splines. Finally, data were visually inspected to check for undetected corrupted segments, and those segments were excluded from the analysis.

Two RS-EEG recordings (1 LU, 1 MV) with less than 100 s of time free of artifacts were excluded from the power analysis. We performed a multitaper spectral analysis to decompose EEG signal into power within the 2–50 Hz range over all electrodes. A 5-s time window, spaced at 1-s intervals, was used to balance temporal resolution and spectral stability. Spectral estimates were computed using a time-bandwidth of 2.5 and four orthogonal tapers [39]. We applied equal weighting to all tapers. Mean log(10) whole-brain power was calculated across the five canonical frequency bands: delta (2–4 Hz), theta (4–6 Hz), alpha (6–13 Hz), beta (13–30 Hz), and gamma (30–50 Hz) [21].

### Statistical analyses

#### Longitudinal analyses

We conducted mixed model analyses to examine developmental, linguistic, and RS-EEG power trajectories across age. Mixed modeling is a successful approach for nested data with varying numbers of time points per participant and is well suited to longitudinal behavioral and neurophysiological research [40, 41]. Separate models were fit for each EEG frequency band. Age and group (TD, LU, LI, MV) were included as fixed effects along with their interaction term, sex as a covariate, and random slopes for age by participant as random effects. Analyses were conducted using the myMixedModelsTrajectories toolbox in MATLAB® R2019b (MathWorks, Natick, MA), fitting linear and quadratic random-slope models to capture different age-related patterns. Behavioral outcomes included Total DQ, EL DQ, RL DQ, FM DQ, VR DQ, and DLPF scores (Vocabulary, Grammar, Pragmatics). RS-EEG power trajectories were estimated across the five frequency bands, and model fit was evaluated using the Bayesian Information Criterion (BIC). In addition to band-limited power analyses, we computed descriptive power spectra for each group (TD, LU, LI, MV), deriving group-level mean spectra with confidence intervals to visualize spectral structure across frequencies.

As the word combination acquisition was a binary categorical variable (i.e., acquired or not), we performed a chi-square test with a 12-month sliding window comparing the proportion of children acquiring combinations at each age bin and applying FDR correction. Moreover, to examine word combination acquisition in relation to whole-brain gamma power, we applied a mixed model analysis fitting linear, quadratic, and cubic random-slope models. The BIC selected the quadratic fit to model gamma power relative to the estimated timing of word combination acquisition. This analysis was limited to the ASD group, as most TD children (63/66, 95.5%) had acquired combinations by their first time point. To compare gamma power before and after acquisition, we excluded autistic children with only one time point (*N* = 48) or two non-consecutive time points (*N* = 6). The final ASD sample included 68 children (41 LU, 18 LI, 9 MV) with 168 RS-EEG recordings (104 LU, 42 LI, 22 MV) between 1.74 and 5.99 years. Nine children (7 MV, 2 LI) out of 68 never acquired word combinations during their participation in our longitudinal protocol. Because the age of acquisition is unknown, these 9 children were considered to have never reached the word combination acquisition stage.

#### Cross-sectional classification analysis

To determine if RS-EEG power features could distinguish between diagnostic groups, we conducted a classification analysis on a cross-sectional sample. The cross-sectional sample (*N* = 188; 122 ASD, 66 TD) was derived from our longitudinal dataset by selecting the first available time point per participant. Of note, for 8 TD participants, the second time point was chosen to better match the older age range of the ASD group. Thus, each participant contributed only one data point, avoiding overlap between training and test sets. Age comparisons indicated no significant difference between the ASD and TD groups (Mann-Whitney U = 3577, *p* = 0.208). However, a small but statistically significant difference across subgroups was observed, as the LI group was slightly older than the TD group (Mann-Whitney U = 1080, *p* = 0.023) and the LU group (Mann-Whitney U = 1035, *p* = 0.046).

We conducted two exploratory classification analyses on the cross-sectional sample (*N* = 188; 122 ASD, 66 TD) to determine if resting-state EEG power features could distinguish between diagnostic groups. The first analysis was a binary classification of ASD versus TD participants. The second was a multi-class classification of three autistic language subgroups and the TD group (a total of four groups). For these analyses, we used 6,400 features derived from the RS-EEG data, comprising power values from 128 electrodes across 5 canonical frequency bands (delta, theta, alpha, beta, gamma) and the first ten 10-s artifact-free time bins. We opted to retain discrete time bins per participant instead of using a single average across time as fluctuations in RS neural activity may contain diagnostically relevant information. While there is no synchronized ‘time course’ across participants, a classifier can leverage these multiple temporal snapshots to learn patterns of intra-individual variability. For instance, the stability or range of power fluctuations across the ten bins may itself be a distinguishing characteristic between groups, information that would be obscured by averaging.

To perform the classifications, we implemented a supervised machine learning pipeline using scikit-learn in Python [42]. This pipeline integrated several steps to ensure a robust evaluation: data scaling, feature selection, and classification with a linear Support Vector Machine (SVM). The entire pipeline was evaluated using a nested cross-validation approach to prevent information leakage and provide an unbiased estimate of model performance. The outer loop of the nested cross-validation used a 5-fold stratified split to divide the data, preserving the percentage of samples for each class. The inner loop employed a 3-fold stratified cross-validation on the training data from the outer loop to perform hyperparameter tuning using a GridSearchCV. Specifically, we optimized the C parameter of the linear SVM (SVC(kernel = ‘linear’)) over a range of values ([0.01, 0.1, 1, 10, 100]). Within this pipeline, we also incorporated a feature selection step using SelectKBest to retain the 500 most informative features based on their ANOVA F-value relative to the diagnostic labels.

To assess the statistical significance of our classification results, we performed a permutation test. For each classification task (2-group and 4-group), we ran the entire nested cross-validation procedure 10 000 times, each time with the diagnostic labels randomly shuffled. This process generated a null distribution of accuracy scores that would be expected by chance. The *p*-value was then calculated as the proportion of permutation-based accuracies that were equal to or greater than the accuracy achieved with the true labels. This rigorous procedure allowed us to determine if the model’s performance was statistically significant. For further model interpretation, we also generated a confusion matrix for each classification to visualize the specific patterns of correct and incorrect predictions across the groups. The matrix is generated from the aggregated out-of-sample predictions from each fold of the outer cross-validation loop, which gives a realistic estimate of performance on unseen data. The code of these analyses can be found at https://github.com/NoCe-Lab/rsEEG_ASD.

## Results

### Description of TD and language profiles

Descriptive analyses of the 66 TD (130 time points) and the 122 ASD (228 time points) participants show no significant age difference at the first available time point (mean age 3.3 ± 1.1 for TD and 3.5 ± 1.0 for ASD) (Table 1). There were significantly more TD females (43.9%) than autistic (12.3%). As expected, autistic participants exhibited higher autism symptom severity, and lower non-verbal and verbal skills. Additionally, there were no significant differences in age at the first timepoint across the LI, LU, MV, and TD groups (*p* > 0.05). As female proportions differed across the four groups, sex was included as a covariate in all analyses. The monolingual *vs*. multilingual environment did not significantly differ. Parental education was higher in TD than in the ASD subgroups (LU, LI, MV). ASD symptom severity and DQs differed across the four groups.

**Fig. 1:**
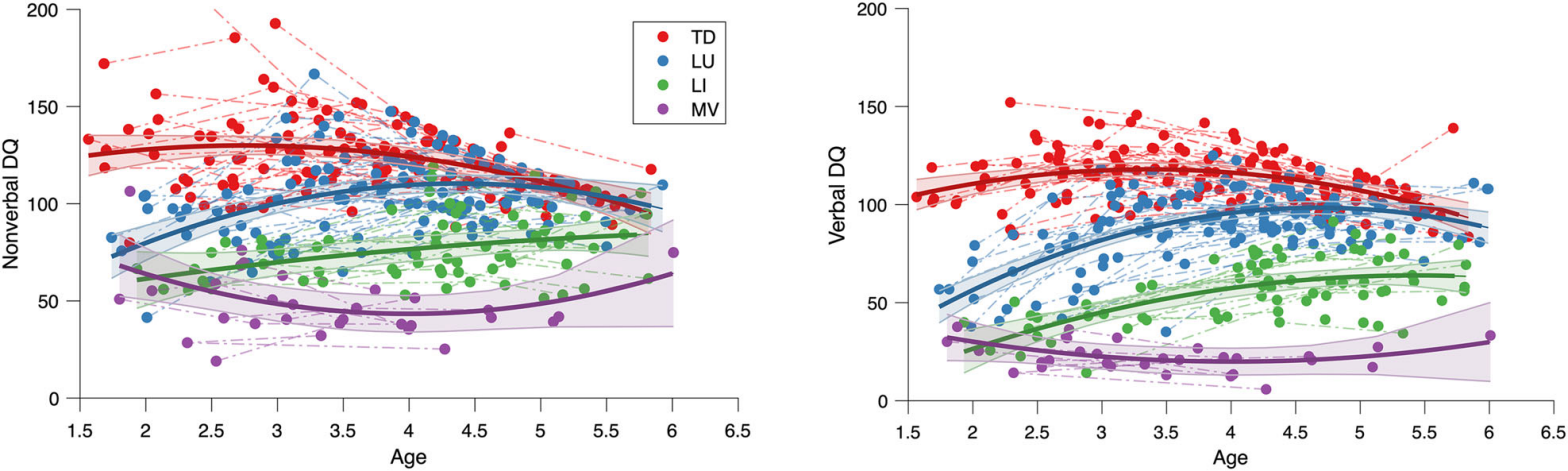
Developmental trajectories across ASD language profiles and TD. Nonverbal and verbal trajectories of the three ASD language profiles and TD group. The colored bands around the estimated group-level trajectory indicate the 95% confidence interval. DQ developmental quotient, TD typical development, LU language unimpaired, LI language impaired, MV minimally-verbal.

To examine developmental trajectories across ASD, TD, and the three language profiles (LU, LI, MV), we analyzed longitudinal changes in non-verbal and verbal skills (Fig. 1). The TD group showed the highest verbal and nonverbal trajectories compared to the three autistic profiles. In comparison, younger LU autistic children exhibited lower verbal and non-verbal skills, though their trajectories fall within the norm later in their development. Children with LI exhibited a more sustained delay in nonverbal and verbal skills, while MV children show limited skills, especially in verbal domains. Additionally, we examined expressive language development with specific measures of their vocabulary, grammar, and pragmatics. Our findings align with our previous work [7], with distinct verbal and non-verbal trajectories within the three language profiles compared to TD children (Supplementary Analysis 1).

### RS-EEG power trajectories: TD vs. ASD

**Fig. 2:**
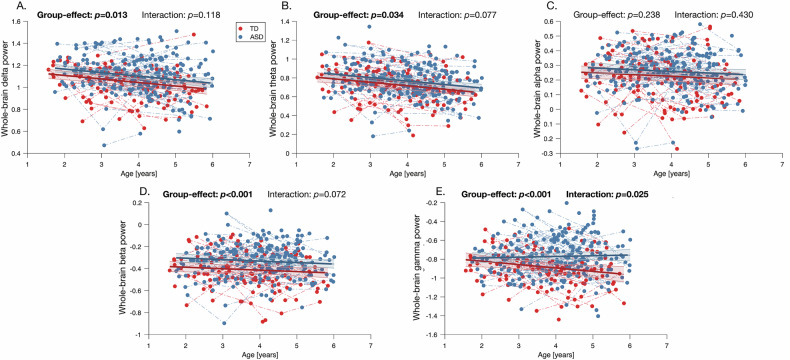
Whole-brain resting-state EEG power trajectories of TD and ASD young children over the five canonical frequency bands. **A** Delta (2–4 Hz). **B** Theta (4–6 Hz). **C** Alpha (6–13 Hz). **D** Beta (13–30 Hz). **E** Gamma (30–50 Hz). The colored bands around the estimated group-level trajectory indicate the 95% confidence interval. TD typical development, ASD autism spectrum disorder.

We compared whole-brain RS-EEG power trajectories of TD and ASD participants over the five frequency bands (Fig. 2, Table S3). In delta, theta, and beta frequency bands, children with ASD showed significantly increased whole-brain power (*p* = 0.013, *p* = 0.034, *p* < 0.001 respectively), with non-significant interactions between band and age. No significant difference was found for whole-brain alpha power. Finally, whole-brain gamma power was significantly higher in the ASD group than in the TD group (*p* < 0.001) and the interaction was also significant (*p* = 0.025), revealing different trajectories over development. While whole-brain gamma power slowly decreased with age in TD children, it remained stable with age in the ASD group.

### RS-EEG power trajectories: TD, LU, LI, and MV

**Fig. 3:**
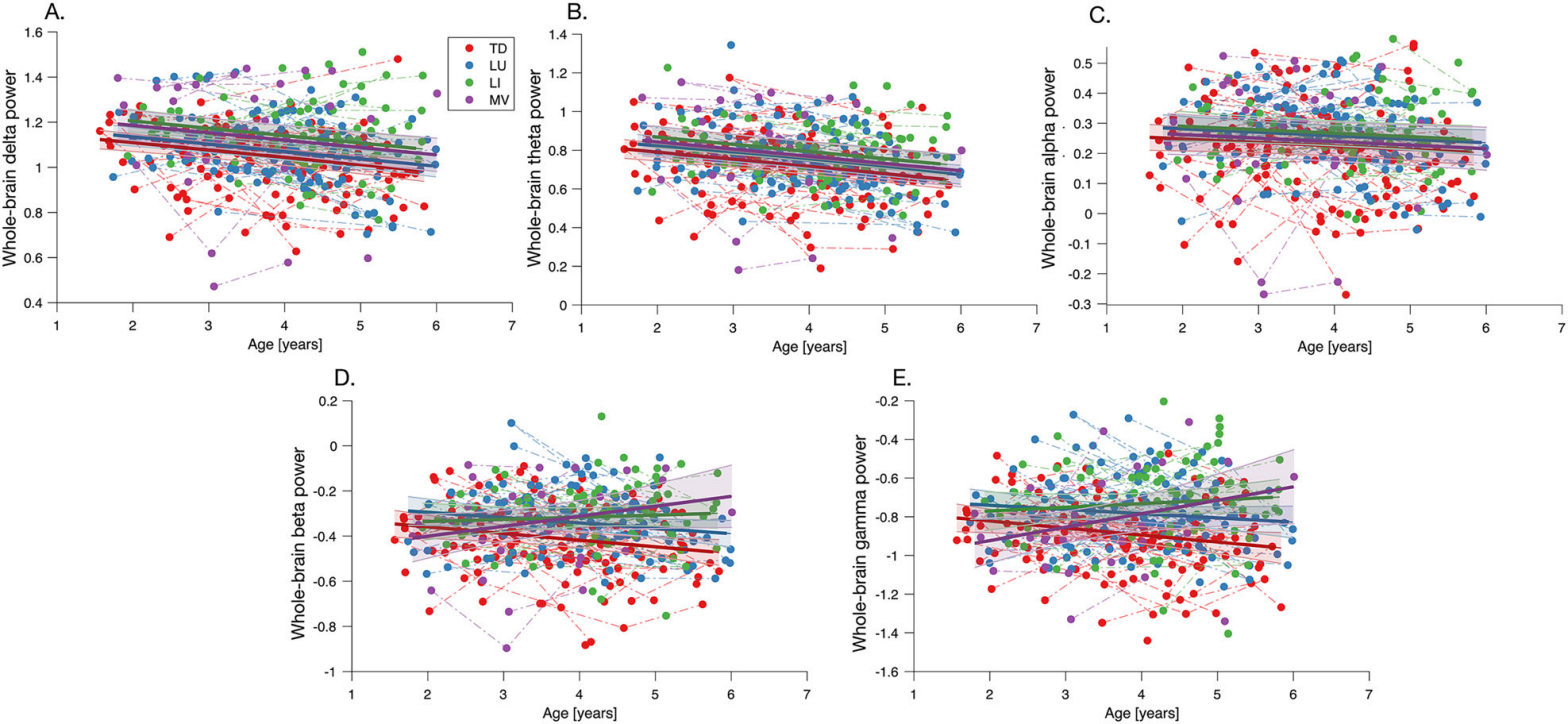
Whole-brain resting-state EEG power trajectories of TD and of the three ASD language profiles over the five canonical frequency bands. **A** Delta (2–4 Hz). **B** Theta (4–6 Hz). **C** Alpha (6–13 Hz). **D** Beta (13–30 Hz). **E** Gamma (30–50 Hz). The colored bands around the estimated group-level trajectory indicate the 95% confidence interval. TD typical development, LU language unimpaired, LI language impaired, MV minimally-verbal.

Building upon the observed differences between the broader ASD and TD groups, we further analyzed whole-brain RS-EEG power trajectories across the three autistic language profiles (LU, LI, MV) (Fig. 3, Table S4). Whole-brain delta trajectories showed significant group effect (*p* = 0.009), but non-significant age-interaction (*p* > 0.05). In both theta and alpha bands, we found no significant group effect or age-interaction (*p* > 0.05). Significant group effect (*p* < 0.001) and age-interaction (*p* = 0.018 and *p* = 0.012, respectively) were observed in beta and gamma bands. While beta and gamma power decreased with age for the TD and the LU groups, it increased over development for LI and MV. Given our hypothesis that whole-brain gamma power differs across the autistic language profiles, we conducted additional analyses comparing the four groups two-by-two. We found significant differences between TD *vs*. LU, TD *vs*. LI, TD *vs*. MV, and LU *vs*. MV (Figure S5, Table S4-5). Group-level power spectra across the groups are provided in Supplementary Figure S6 for descriptive comparison.

### RS-EEG gamma power and word combination acquisition

**Fig. 4:**
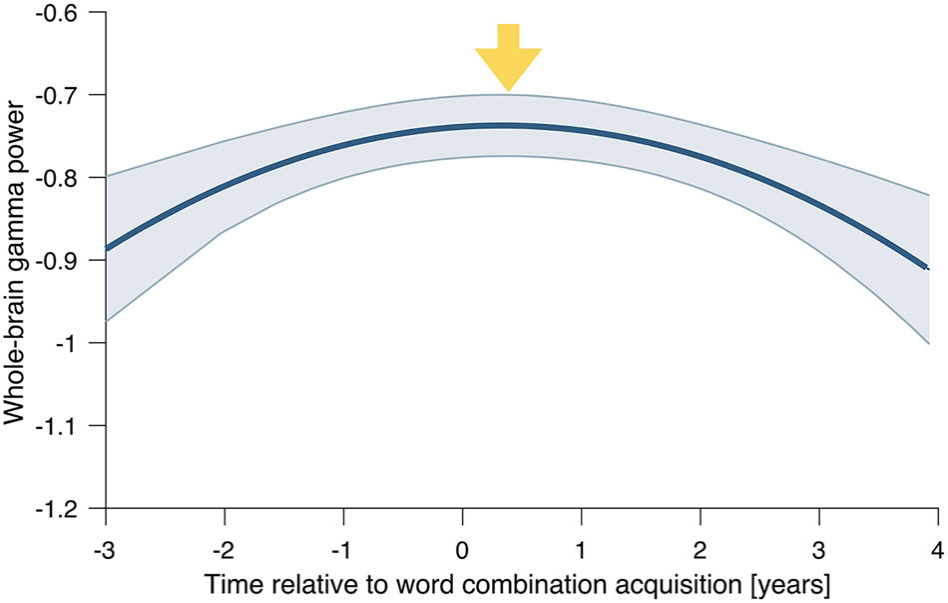
Trajectory of whole-brain resting-state EEG gamma power trajectory of the ASD group (*N* = 68, 168 time points), aligned with the age of word combination acquisition. The x-axis represents time relative to individual word combination acquisition (in years). Age of word combination acquisition, or time 0, is marked by the dashed vertical line. The solid black line shows the estimated group-level trajectory, and the shaded area represents the 95% confidence interval. The yellow arrow indicates the peak of the curve, which was calculated at 0.384 years, i.e., approximately 4 months post-word combination acquisition. Whole-brain gamma power then decreases after acquisition of this milestone.

Considering the heterogeneity in word combination acquisition and the association between gamma power and language in ASD [21, 23], we examined the trajectory of gamma power in our ASD sample. The quadratic model showed a progressive increase in gamma power, before plateauing near phrase speech acquisition (Fig. 4), with the peak occurring 0.384 years (approximately 4 months) post-acquisition. Gamma power then decreased after acquisition, suggesting its crucial role in the transition from single words to phrases. To assess the robustness of this finding, we conducted an additional mixed-model analysis omitting the nine children who never acquired word combinations (*N* = 59, 146 time points). This analysis also yielded a quadratic fit, with the peak slightly shifted to 0.783 years (approximately 9 months) post-acquisition (Figure S7).

### RS-EEG power features-based classifications

#### Binary Classification: ASD vs. TD

The supervised machine learning pipeline distinguished between individuals with ASD and TD participants with a mean accuracy of 69.2%. A permutation test, which involved running the entire cross-validation procedure 10 000 times with shuffled labels, confirmed that this accuracy was highly significant (*p* = 0.0004). The observed true accuracy was well beyond the distribution of accuracies obtained from the permuted data, as illustrated in the permutation plot (Figure S8). The confusion matrix from the cross-validation reveals the specific performance of the classifier:

- Sensitivity (ASD identification): The model correctly identified 94 of the 122 individuals with ASD, yielding a sensitivity of 77.0%.
- Specificity (TD identification): The model correctly identified 36 of the 66 TD individuals, for a specificity of 54.5%.

#### Multi-Class Classification: 4 Language Profiles

For the four-group classification task (distinguishing between the three ASD language subgroups and the TD group), the model achieved a mean accuracy of 44.6%. This performance is substantially higher than the chance level of 25%. The permutation test demonstrated that this result was highly statistically significant (*p* < 0.0001), with the true accuracy falling far outside the null distribution. An analysis of the confusion matrix shows that classification performance varied considerably across the four groups:

- The model was most successful in identifying TD participants, classifying correctly 66.7% of cases (44 out of 66).
- The model performed poorly in identifying the ASD-MV group, with only 1 correct classification out of 17 cases (5.9% accuracy).
- There was considerable confusion between the ASD-LI and ASD-LU subgroups, which the model often misclassified as each other (respectively 40.90 and 34.4% of accuracy).

## Discussion

In this study, we investigated whole-brain RS-EEG power trajectories in a substantial longitudinal sample of 188 children (*N* = 122 ASD, *N* = 66 TD), yielding 358 time points. Given the heterogeneous language abilities in autism, we explored RS-EEG power trajectories among three autistic language profiles: Language Unimpaired (LU, *N* = 61), Language Impaired (LI, *N* = 44), and Minimally-Verbal (MV, *N* = 17). We provide valuable insights into the neural correlates of language development in autism.

In line with our hypothesis, our RS-EEG power trajectories support the U-shaped profile previously reported in autism (Fig. 2) [13]. The ASD group exhibited increased power in low-frequency bands (delta, theta), high-frequency bands (beta, gamma), and similar alpha power compared to the TD group. While the U-shaped profile has been corroborated [13, 18, 19], findings have been inconsistent. Some evidence suggests that enhanced delta power in ASD is especially different in lower functioning children [13, 14, 43]. This finding aligns with our LI group showing significantly higher delta power than TD peers. However, other studies reported enhanced delta power in high functioning children [13, 15]. Although a meta-analysis found no difference compared to TD participants [18], we observed increased theta power in autistic children, consistent with previous studies [15, 16]. Additionally, our results align with the established decline of delta and theta power with age [17], reflecting brain maturation, gray matter tissue loss, and increased processing efficiency [44]. While reduced alpha power in ASD has been proposed as a biomarker [18, 44], our findings and those from large cohort studies [17] do not support this. Findings on beta power in ASD are inconsistent with some meta-analyses reporting both non-significant differences and increased power [13, 17, 18].

We show that fine-grained RS-EEG features can distinguish TD from autistic participants and, to a degree, between language-based subgroups within the autism spectrum. Our binary classification of ASD versus TD achieved a statistically significant accuracy of 69.2% (*p* < 0.0014), establishing that EEG signatures can differentiate these groups. The more complex multi-class model also performed significantly above chance, achieving 44.6% accuracy in differentiating the four language profiles (*p* < 0.0001). Despite this statistical significance, the practical utility of these models must be carefully considered. The confusion matrices reveal challenges that preclude immediate clinical application; for instance, the binary classifier had greater sensitivity in identifying ASD participants than specificity in identifying TD participants. Furthermore, the multi-class model showed inconsistent performance, struggling to accurately classify certain ASD subgroups, particularly the MV profile. Consequently, while these findings provide a robust proof-of-concept for the utility of RS-EEG in characterizing neurodevelopmental heterogeneity, the current models are not sufficiently robust to support individual-level diagnostic use. This conclusion is consistent with previous reviews indicating that EEG currently lacks the sensitivity and specificity required for clinical diagnosis in autism [45–47]. Future studies are needed to explore the full RS-EEG power spectrum in ASD, by using larger samples that span early childhood to adulthood and include varying levels of functioning.

As expected, autistic children showed increased gamma compared to TD peers. Autistic children with LU exhibited greater gamma power than TD children (Figure S6). However, LU trajectories were more similar to TD than those of LI and MV children, who showed more divergent patterns (Figure S6). These results align with previous frontal EEG studies on autistic children [23] and on HL-infants later diagnosed with ASD [21], and our whole-brain approach extends their findings. Furthermore, since gamma oscillations might reflect the balance between E/I (or glutamatergic/GABAergic) systems, globally reduced gamma power may indicate compensatory mechanisms to early aberrant neurocircuitry, potentially supporting language development in young autistic children and HL-infant siblings later diagnosed [21–23, 48, 49]. Future research could test this hypothesis by investigating aperiodic power, as it may reflect the E/I balance in the brain [22]. Given the close relationship between gamma power and the aperiodic component of the EEG spectrum, future work should aim to disentangle periodic and aperiodic contributions to determine their respective roles in language development. Moreover, combining EEG with neurochemical imaging techniques such as Magnetic Resonance Spectroscopy could further elucidate the role of glutamatergic and GABAergic systems as compensatory processes in language development.

Taken together, our results highlight the relevance of gamma oscillations to language abilities in ASD. Our analysis of word combination acquisition revealed a complex developmental pattern: gamma power in ASD increased prior to this milestone, peaked near acquisition, and subsequently declined. This trajectory suggests that an initial increase in gamma power (reflecting heightened neural activity [48–50]) followed by a decline may support the emergence of phrase speech in ASD. The subsequent decline in gamma power, potentially reflecting diminished excitation, may indicate more efficient language and cognitive processing, as previously proposed [21, 23, 49, 50]. This two-phase increase-decrease process appears critical, as persistently increasing gamma power trajectories throughout early childhood in the MV group were not associated with greater language gains or word combination acquisition. To our knowledge, our study is the first to link RS-EEG gamma power to word combination. This finding needs replication in autistic children who reach this milestone after age 5 [51, 52], as well as in children with developmental language disorder and TD children. In our sample, early acquisition of this milestone by TD children limited opportunities for comparison. Nonetheless, this finding may reflect neural mechanisms underlying both autistic language trajectories and the transition from single words to phrase speech, a critical step toward functional language [36].

Collecting and analyzing neural signals in typical and atypical early development presents significant challenges due to the dynamic interplay of age, developmental variability and clinical heterogeneity. Our study reflects a considerable effort to carefully disentangle these factors through a robust longitudinal design, enabling RS-EEG analysis across distinct autistic language profiles. However, several limitations should be acknowledged. While our adapted RS-EEG paradigm (recorded during a non-silent cartoon) was necessary to ensure engagement and obtain good-quality data from young participants, it differs from typical resting-state protocols involving silent videos. While necessary for participant engagement and data quality, it deviates from standard RS-EEG protocols and should be considered when interpreting the results. Even though we provided materials to help children and their parents familiarize themselves with the EEG, the data collection was not successful for all children. A minority of MV children tolerated the EEG net, which highlights the need for specific habituation procedures for RS-EEG acquisition [16]. Considering the variability within MV children, especially in non-verbal and receptive language skills [12], a larger MV group could provide a more detailed understanding of gamma power differences. It will also be important to follow MV children into school-age, as some may develop phrase speech later [12]. As our longitudinal cohort was recently extended to school-age [53], future work will allow us to address these questions, including whether some individuals transition between language profiles after 6 years of age [54]. Finally, although RS-EEG features-based classification showed some promise for distinguishing clinical groups, our findings remain only preliminary and are not yet clinically applicable. The use of age-matched TD and autistic groups limited our ability to disentangle purely developmental effects from ASD-specific neural characteristics. Future studies could therefore incorporate demographic variables, such as age, directly into classification models. Moreover, this work could be extended by including additional EEG features, such as functional connectivity and aperiodic activity, which may provide a more comprehensive characterization of neurodevelopmental heterogeneity.

In conclusion, our longitudinal investigation of 122 autistic and 66 TD young children provides evidence for distinct RS-EEG power trajectories, partially supporting the U-shaped spectral profile in autism [13]. Examining three validated autistic language profiles (LU, LI, MV) represents a significant strength of our study, revealing how gamma power trajectories vary within ASD and closely align with language abilities, thereby positioning gamma power as a potential marker of language heterogeneity. Gamma power followed a quadratic trajectory around word combination acquisition, indicating a compensatory neural mechanism [21, 23] facilitating the transition to phrase speech. As phrase speech is a critical milestone toward functional language and positive outcomes for autistic individuals (e.g., improved quality of life and independence) [3, 36, 55], early language development should remain a priority in intervention efforts. Further understanding of the underlying neural mechanisms may inform the development of effective early intervention approaches.

## Supplementary information

Supplementary Material

## Data Availability

The datasets analyzed during the current study are not publicly available due to privacy and ethical restrictions but are available from the corresponding author upon reasonable request.
