# Supplementary material for: Early Trajectories of Resting-State EEG power in autistic children: a longitudinal study across language profiles
Source: Transl Psychiatry. 2026 May 25;16:371. doi: 10.1038/s41398-026-04132-0 (PMC13385626; doi:10.1038/s41398-026-04132-0)
Supplement: Supplementary file 1 — Supplementary Material [file 41398_2026_4132_MOESM1_ESM.docx]

# **Supplementary Materials**

## **Supplementary Analysis 1 :** Longitudinal development of verbal, non-verbal, and expressive language skills

By conducting mixed model analyses, we investigated the longitudinal trajectories of verbal (receptive and expressive language) and non-verbal (visual reception and fine motor) skills in the ASD and TD groups, and in the three language profiles. Moreover, we examined the development of expressive language using fine-grained measures of vocabulary, grammar, and pragmatics. Although the sample sizes are smaller than in our previous work^1^ due to selecting only children with available RS-EEGs, our results remain consistent. The ASD group globally showed lower verbal and non-verbal trajectories, and lower expressive language skills compared to the TD group (*p*<0.001, Figures S1-2, Table S1). In addition, children belonging to the LU profile exhibited higher verbal and non-verbal trajectories compared those in the LI and MV profiles. Children within the LI profile showed higher scores than MV children (Figure S3, Table S2). Distinct vocabulary, grammar, pragmatics, and word combination acquisition trajectories were found in LU, LI, MV^1^ . TD children produced an average of 1104 words, compared to 852.6 words in LU, 454.7 words in LI, and 24.4 words in MV. Delayed vocabulary emergence was observed even in LU, while the LI group showed both delayed and slowed development, and MV had minimal acquisition (Figure S4a, Table S2). Furthermore, over 75% of TD children acquired word combinations by age 2, reaching 100% by 2.5 years. In the LU group, ~50% reached this milestone by age 2, and nearly all by age 3.5. In the LI group, ~25% acquired this skill by age 3.5, rising to ~75% by age 4. The MV group displayed the greatest delays, with ~25% by age 5, and ~50% by age 6 (Figure S4d, Table S2).

**
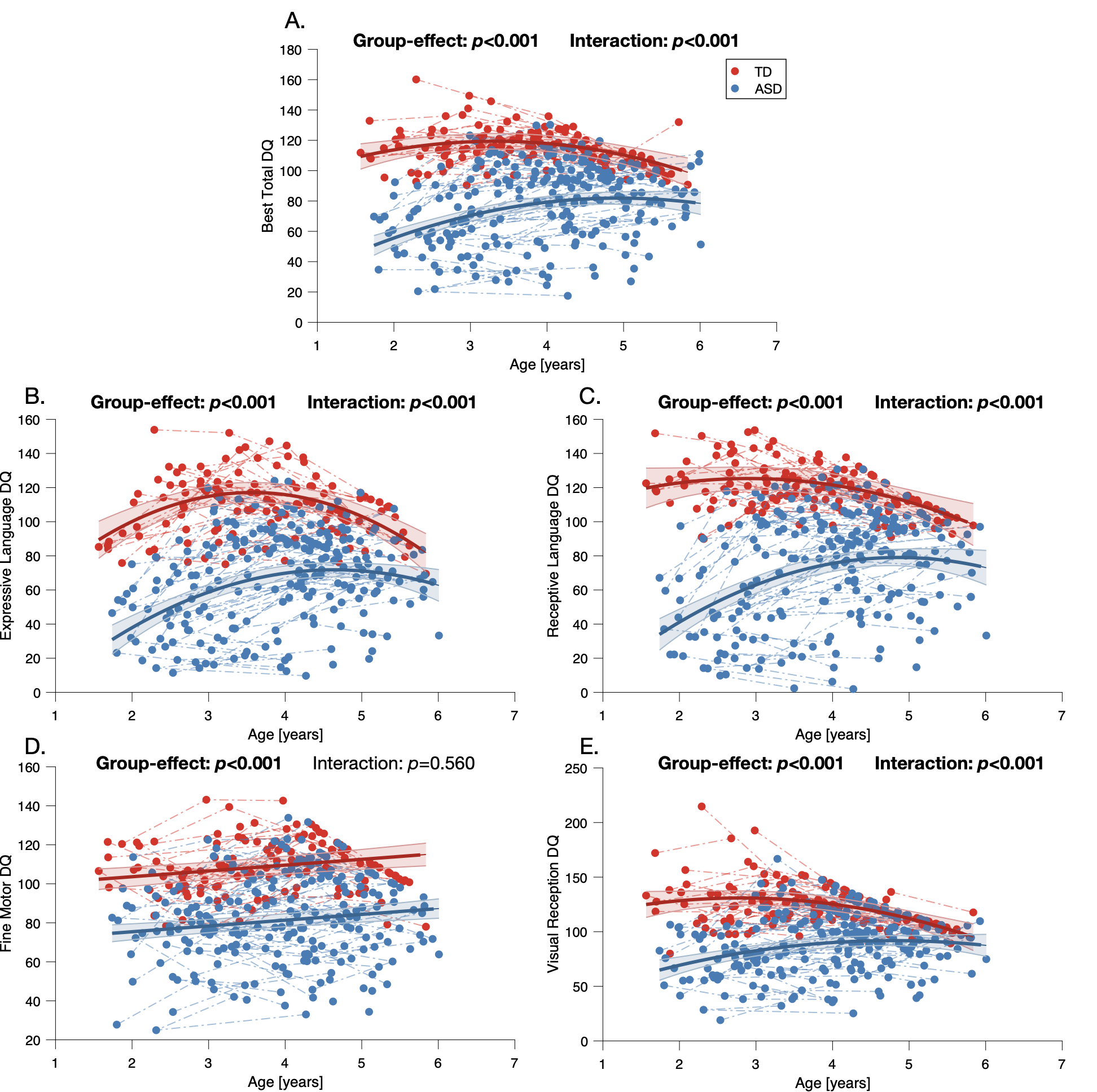
**

**Figure S1.** Developmental trajectories of TD and ASD young children. **A.** Total DQ (average of all domains). **B.** Expressive Language DQ. **C.** Receptive Language DQ. **D.** Fine Motor DQ. **E.** Visual Reception DQ.

The colored bands around the estimated group-level trajectory indicate the 95% confidence interval. DQ: developmental quotient ; TD: typical development ; ASD: autism spectrum disorder.

**
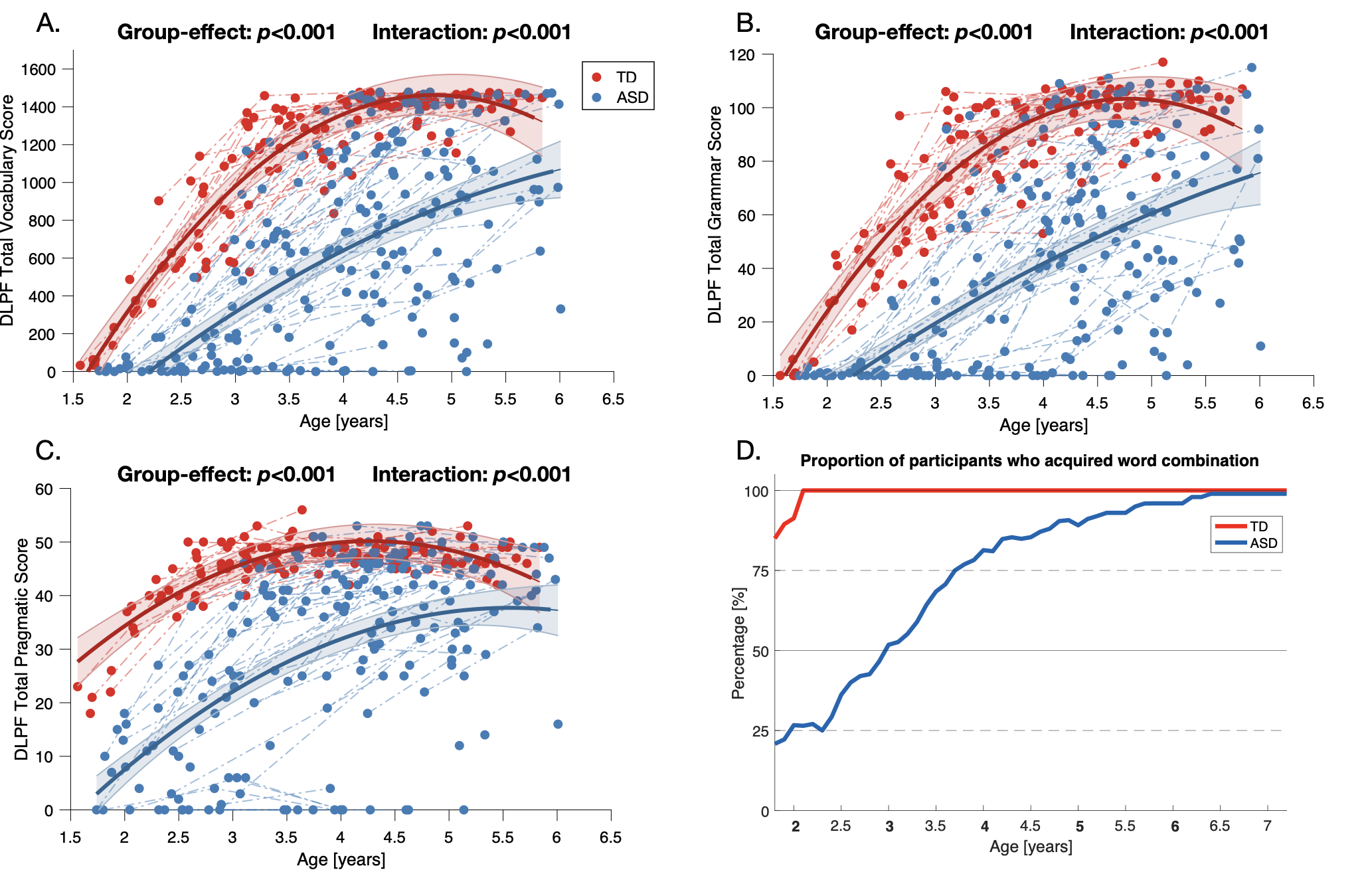
**

**Figure S2.** Extensive expressive language trajectories of TD and ASD young children.
**A.** DLPF total vocabulary score **B.** DLPF total grammar score. **C.** DLPF total pragmatic score. **D.** DLPF proportion of word combination.

The colored bands around the estimated group-level trajectory indicate the 95% confidence interval. DLPF: *questionnaire du développement du langage productif en français* ; TD: typical development ; ASD: autism spectrum disorder.

**Table S1**. Results of the mixed model analysis comparing developmental and linguistic skills between the TD and ASD groups. Note: P-value group effect evaluates the difference between mean scores in the two groups whereas p-value interaction refers to the difference in the shape of the developmental trajectory. The significance threshold was fixed at 0.05, and statistically significant values are shown in bold.

TD = Typical development; ASD = Autism spectrum disorder; DQ = Developmental Quotient. DLPF = *Développement du langage productif en français*.

| **TD vs. ASD** | **Group effect** | | | | | **Interaction with age** | | | |
| --- | --- | --- | --- | --- | --- | --- | --- | --- | --- |
|  | Model order | *p-*value | ASD intercept (± SD) | TD intercept  (± SD) | Log likelihood, df | *p*-value | ASD age slope (± SD) | TD  age slope (± SD) | Log likelihood, df |
| **Total DQ** | quadratic | **<0.001** | 7.50 ± 9.82 | 83.52 ± 11.32 | 161.52, 3 | **<0.001** | 30.15 ± 5.24 | 21.58 ± 6.33 | 42.75, 2 |
| **Visual Reception DQ** | quadratic | **<0.001** | 25.14 ± 14.71 | 100.32 ± 16.63 | 116.46, 3 | **<0.001** | 27.92 ± 7.91 | 21.82 ± 9.30 | 39.96, 2 |
| **Fine Motor DQ** | linear | **<0.001** | 68.16 ± 3.95 | 99.62 ± 4.82 | 76.35, 2 | 0.560 | 3.33 ± 1.00 | 2.38 ± 1.27 | 0.34, 1 |
| **Receptive Language DQ** | quadratic | **<0.001** | -30.17 ± 14.33 | 98.99 ± 16.15 | 168.62, 3 | **<0.001** | 44.89 ± 7.71 | 18.14 ± 9.02 | 56.92, 2 |
| **Expressive Language DQ** | quadratic | **<0.001** | -33.01 ± 13.39 | 29.37 ± 15.13 | 158.35, 3 | **<0.001** | 45.21 ± 7.20 | 49.13 ± 8.45 | 33.31, 2 |
| **DLPF Total Vocabulary Score** | quadratic | **<0.001** | -1116.43 ± 178.91 | -1867.95 ± 194.38 | 142.05, 3 | **<0.001** | 589.88 ± 98.81 | 1375.89 ± 114.23 | 24.81, 2 |
| **DLPF Total Grammar Score** | quadratic | **<0.001** | -69.30 ± 14.57 | -131.07 ± 16.19 | 139.23, 3 | **<0.001** | 35.02 ± 8.07 | 97.99 ± 9.49 | 23.29, 2 |
| **DLPF Total Pragmatic Score** | quadratic | **<0.001** | -35.79 ± 5.50 | -5.98 ± 6.59 | 127.07, 3 | **<0.001** | 26.40 ± 3.04 | 26.32 ± 3.74 | 34.85, 2 |

**
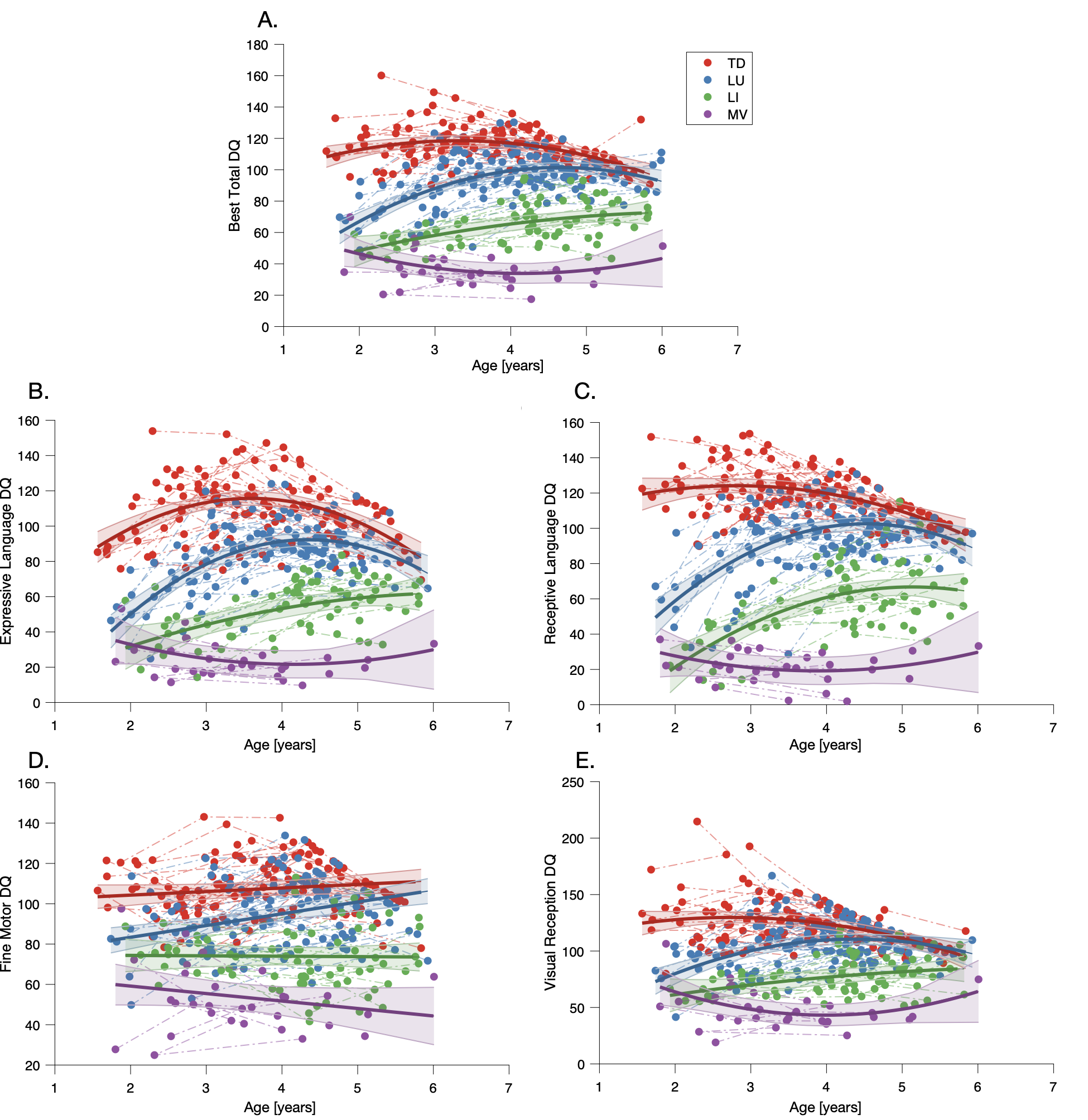
**

**Figure S3.** Developmental trajectories of the three ASD language profiles. A. Total DQ (average of all domains). B. Expressive Language DQ. C. Receptive Language DQ. D. Fine Motor DQ. E. Visual Reception DQ.

The colored bands around the estimated group-level trajectory indicate the 95% confidence interval. DQ: developmental quotient ; TD: typical development ; LU: language unimpaired; LI: language impaired; MV: minimally-verbal.

**
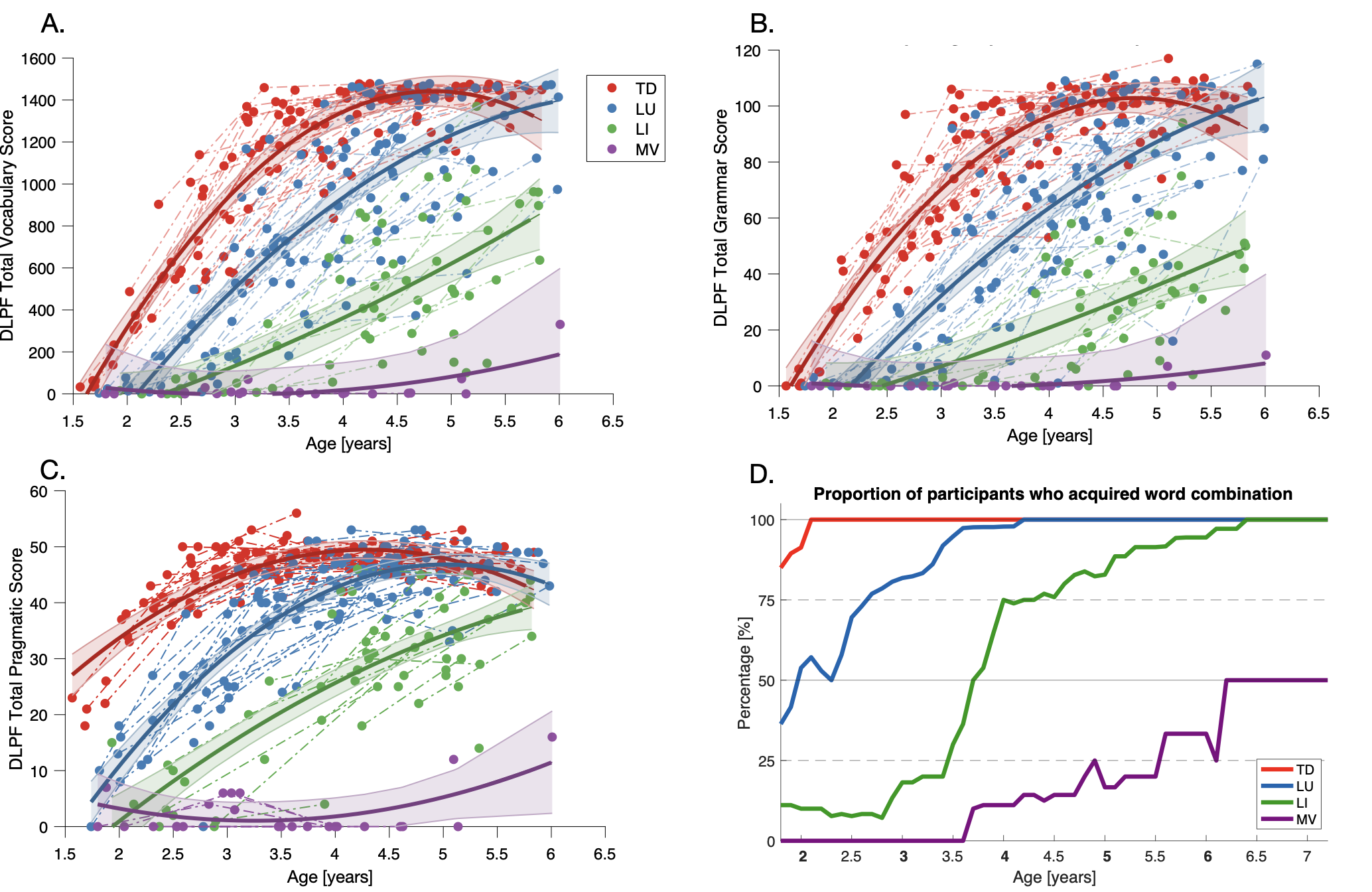
**

**Figure S4.** Extensive expressive language trajectories of the three ASD language profiles. **A.** DLPF total vocabulary score **B.** DLPF total grammar score. **C.** DLPF total pragmatic score. **D.** DLPF proportion of word combination.

The colored bands around the estimated group-level trajectory indicate the 95% confidence interval. DLPF: *questionnaire du développement du langage productif en français*; TD: typical development; LU: language unimpaired; LI: language impaired; MV: minimally-verbal.

**Table S2.** Results of the mixed model analysis comparing developmental and linguistic skills between TD and the three ASD language profiles. Note: P-value group effect evaluates the difference between mean linguistic scores in the groups whereas p-value interaction refers to the difference in the shape of the linguistic trajectory. The significance threshold was fixed at 0.05, and statistically significant values are shown in bold.

LU = Language Unimpaired; LI = Language Impaired; MV = Minimally-Verbal; DQ = Developmental Quotient. DLPF = Développement du langage productif en français.

| **LU vs. LI vs. MV** |  | | **Group effect** | | | | **Interaction with age** | | | | |
| --- | --- | --- | --- | --- | --- | --- | --- | --- | --- | --- | --- |
|  | Model order | *p-*value | LU intercept (± SD) | LI intercept  (± SD) | MV intercept (± SD) | Log likelihood, df | *p*-value | LU age slope (± SD) | LI  age slope (± SD) | MV  age slope  (± SD) | Log likelihood, df |
| **Total DQ** | quadratic | **<0.001** | 79.38 ± 20.42 | 23.28 ± 18.77 | -4.69 ± 11.07 | 192.71, 6 | **<0.001** | -56.10 ± 27.81 | -19.51 ± 13.11 | 11.10 ± 21.68 | 46.01, 4 |
| **Visual Reception DQ** | quadratic | **<0.001** | 125.81 ± 30.10 | 39.46 ± 27.39 | 6.70 ± 17.85 | 123.42, 6 | **<0.001** | -86.35 ± 40.82 | -32.36 ± 19.25 | 2.29 ± 32.06 | 27.91, 4 |
| **Fine Motor DQ** | linear | **<0.001** | 66.81 ± 9.48 | 74.80 ± 7.33 | 71.27 ± 5.35 | 84.77, 6 | **<0.001** | 7.99 ± 11.94 | 11.40 ± 9.13 | 17.53 ± 10.28 | 13.78, 4 |
| **Receptive Language DQ** | quadratic | **<0.001** | 53.81 ± 28.54 | -50.98 ± 25.73 | -37.89 ± 17.16 | 192.47, 6 | **<0.001** | -104.79 ± 38.55 | -42.48 ± 18.19 | -25.83 ± 30.26 | 31.06, 4 |
| **Expressive Language DQ** | quadratic | **<0.001** | 64.90 ± 23.98 | -25.98 ± 15.30 | -51.79 ± 14.37 | 198.11, 6 | **<0.001** | -67.80 ± 32.42 | -25.98 ± 15.30 | 19.74 ± 25.46 | 47.13, 4 |
| **DLPF Total Vocabulary Score** | quadratic | **<0.001** | 182.49 ± 467.76 | -337.47 ± 375.90 | -1557,81 ± 210.36 | 127.31, 6 | **<0.001** | -519.96 ± 604.58 | -295.62 ± 273.99 | 484.85 ± 444.01 | 62.77, 4 |
| **DLPF Total Grammar Score** | quadratic | **<0.001** | -1.99 ± 12.40 | -36.06 ± 10.28 | -58.11 ± 6.45 | 116.00, 6 | **<0.001** | -34.08 ± 16.08 | -20.59 ± 12.10 | -5.47 ± 13.82 | 54.08, 4 |
| **DLPF Total Pragmatic Score** | quadratic | **<0.001** | 15.04 ± 11.48 | -34.51 ± 11.33 | -51.99 ± 6.40 | 202.61, 6 | **<0.001** | -49.55 ± 16.16 | -20.61 ± 7.69 | -1.61 ± 12.91 | 73.19, 4 |

**Bibliography**

1. Latrèche, K. *et al.* Early trajectories and moderators of autistic language profiles: A longitudinal study in preschoolers. *Autism* 13623613241253015 (2024) doi:10.1177/13623613241253015.

**Supplementary Tables**

**Table S3**. Results of the mixed model analysis comparing resting-state EEG power between the TD and ASD groups. Note: P-value group effect evaluates the difference between power in the two groups whereas p-value interaction refers to the difference in the shape of the resting-state EEG power trajectory. The significance threshold was fixed at 0.05, and statistically significant values are shown in bold.

TD = Typical development; ASD = Autism spectrum disorder.

| **TD vs. ASD** | **Group effect** | | | | | **Interaction with age** | | | |
| --- | --- | --- | --- | --- | --- | --- | --- | --- | --- |
|  | *Model order* | *p-*value | ASD intercept (± SD) | TD intercept  (± SD) | Log likelihood, df | p-value | ASD age slope (± SD) | TD  age slope (± SD) | Log likelihood, df |
| **Whole-brain delta power** | linear | **0.013** | 1.20 ± 0.03 | 1.21 ± 0.04 | 8.65, 2 | 0.118 | -0.02 ± 0.01 | -0.04 ± 0.01 | 2.45, 1 |
| **Whole-brain theta power** | linear | **0.034** | 0.88 ± 0.03 | 0.91 ± 0.04 | 6.76, 2 | 0.077 | -0.03 ± 0.01 | -0.05 ± 0.01 | 3.13, 1 |
| **Whole-brain alpha power** | linear | 0.238 | 0.29 ± 0.03 | 0.29 ± 0.04 | 2.87, 2 | 0.430 | -0.01 ± 0.01 | -0.02 ± 0.01 | 0.62, 1 |
| **Whole-brain beta power** | linear | **<0.001** | -0.32 ± 0.04 | -0.30 ± 0.05 | 16.14, 2 | 0.072 | -0.00 ± 0.01 | -0.03 ± 0.01 | 3.24, 1 |
| **Whole-brain gamma power** | linear | **<0.001** | -0.80 ± 0.05 | -0.75 ± 0.06 | 18.30, 2 | **0.025** | 0.01 ± 0.01 | -0.04 ± 0.02 | 5.05, 1 |

**Table S4**. Results of the mixed model analysis comparing resting-state EEG power between the TD and the three ASD language profiles. Note: P-value group effect evaluates the difference between power in the two groups whereas p-value interaction refers to the difference in the shape of the resting-state EEG power trajectory. The significance threshold was fixed at 0.05. P-values are adjusted for false discovery rate (FDR); bold values indicate statistically significant results.

TD = Typical development; LU = Language Unimpaired; LI = Language Impaired; MV = Minimally-Verbal.

| **TD vs. LU vs. LI vs. MV** | **Group effect** | | | | | | | **Interaction with age** | | | | | |
| --- | --- | --- | --- | --- | --- | --- | --- | --- | --- | --- | --- | --- | --- |
|  | Model order | *p-*value | MV intercept (± SD) | TD intercept (± SD) | LU intercept  (± SD) | LI intercept (± SD) | Log likelihood, df | p-value | MV  age slope (± SD) | TD age slope (± SD) | LU  age slope (± SD) | LI  age slope  (± SD) | Log likelihood, df |
| **Whole-brain delta power** | linear | 0.009 | 1.16 ± 0.08 | 1.21 ± 0.04 | 1.21 ± 0.04 | 1.20 ± 0.06 | 17.00, 6 | 0.206 | 0.05 ± 0.09 | 0.01 ± 0.07 | 0.02 ± 0.07 | 0.04 ± 0.08 | 4.58, 3 |
| **Whole-brain theta power** | linear | 0.103 | 0.88 ± 0.09 | 0.92 ± 0.04 | 0.90 ± 0.05 | 0.84 ± 0.07 | 10.56, 6 | 0.175 | 0.02 ± 0.10 | -0.00 ± 0.08 | 0.01 ± 0.08 | 0.04 ± 0.08 | 4.95, 3 |
| **Whole-brain alpha power** | linear | 0.451 | 0.24 ± 0.08 | 0.29 ± 0.04 | 0.33 ± 0.05 | 0.23 ± 0.06 | 5.76, 6 | 0.391 | 0.09 ± 0.10 | 0.07 ± 0.08 | 0.07 ± 0.07 | 0.10 ± 0.08 | 3.01, 3 |
| **Whole-brain beta power** | linear | **<0.001** | -0.49 ± 0.09 | -0.30 ± 0.05 | -0.25 ± 0.05 | -0.35 ± 0.07 | 24.30, 6 | **0.018** | 0.24 ± 0.11 | 0.17 ± 0.09 | 0.17 ± 0.08 | 0.21 ± 0.09 | 10.11, 3 |
| **Whole-brain gamma power** | linear | **<0.001** | -1.06 ± 0.11 | -0.75 ± 0.05 | -0.70 ± 0.06 | -0.80 ± 0.09 | 29.19, 6 | **0.012** | 0.37 ± 0.13 | 0.26 ± 0.11 | 0.27 ± 0.10 | 0.31 ± 0.11 | 10.93, 3 |

|  | **TD vs. LU** | | | | | | | | | **TD vs. LI** | | | | | | | | | **TD vs. MV** | | | | | | | | |
| --- | --- | --- | --- | --- | --- | --- | --- | --- | --- | --- | --- | --- | --- | --- | --- | --- | --- | --- | --- | --- | --- | --- | --- | --- | --- | --- | --- |
|  | **Group effect** | | | | | **Interaction with age** | | | | **Group effect** | | | | | **Interaction with age** | | | | **Group effect** | | | | | **Interaction with age** | | | |
|  | Model order | *p*-value | TD intercept  (± SD) | LU intercept  (± SD) | Log likelihood, df | *p*-value | TD age slope (± SD) | LU  age slope (± SD) | Log likelihood, df | Model order | *p*-value | TD intercept  (± SD) | LI intercept  (± SD) | Log likelihood, df | *p*-value | TD age slope (± SD) | LI  age slope (± SD) | Log likelihood, df | Model order | *p*-value | MV intercept  (± SD) | LU intercept  (± SD) | Log likelihood, df | *p*-value | MV  age slope (± SD) | LU age slope (± SD) | Log likelihood, df |
| **Whole-brain delta power** | linear | 0.280 | 1.21 ± 0.04 | 1.22 ± 0.04 | 2.54, 2 | 0.622 | -0.05 ± 0.01 | -0.04 ± 0.01 | 0.24, 1 | linear | **<0.001** | 1.20 ± 0.04 | 1.19 ± 0.06 | 16.91, 2 | 0.076 | -0.04 ± 0.01 | -0.01 ± 0.01 | 3.15, 1 | linear | 0.135 | 1.16 ± 0.09 | 1.21 ± 0.04 | 4.00, 2 | 0.157 | 0.00 ± 0.02 | -0.03 ± 0.01 | 2.01, 1 |
| **Whole-brain theta power** | linear | 0.332 | 0.91 ± 0.04 | 0.91 ± 0.05 | 2.21, 2 | 0.424 | -0.05 ± 0.01 | -0.04 ± 0.01 | 0.64, 1 | linear | 0.009 | 0.91 ± 0.04 | 0.84 ± 0.07 | 9.49, 2 | 0.052 | -0.05 ± 0.01 | -0.01 ± 0.02 | 3.78, 1 | linear | 0.701 | 0.88 ± 0.08 | 0.88 ± 0.04 | 0.71, 2 | 0.604 | -0.02 ± 0.02 | -0.04 ± 0.01 | 0.27, 1 |
| **Whole-brain alpha power** | linear | 0.217 | 0.29 ± 0.04 | 0.33 ± 0.05 | 3.06, 2 | 0.936 | -0.02 ± 0.01 | -0.02 ± 0.02 | 0.01, 1 | linear | 0.104 | 0.29 ± 0.04 | 0.23 ± 0.07 | 4.53, 2 | 0.175 | -0.02 ± 0.01 | 0.01 ± 0.02 | 1.84, 1 | linear | 0.636 | 0.25 ± 0.08 | 0.33 ± 0.04 | 0.91, 2 | 0.347 | 0.00 ± 0.02 | -0.02 ± 0.01 | 0.88, 1 |
| **Whole-brain beta power** | linear | **0.003** | -0.31 ± 0.05 | -0.25 ± 0.05 | 11.42, 2 | 0.706 | -0.03 ± 0.01 | -0.02 ± 0.01 | 0.14, 1 | linear | **<0.001** | -0.31 ± 0.05 | -0.36 ± 0.07 | 14.80, 2 | 0.059 | -0.03 ± 0.01 | 0.01 ± 0.02 | 3.58, 1 | linear | 0.033 | -0.47 ± 0.09 | -0.25 ± 0.05 | 6.85, 2 | **0.010** | 0.04 ± 0.02 | -0.02 ± 0.01 | 6.73, 1 |
| **Whole-brain gamma power** | linear | **<0.001** | -0.76 ± 0.06 | -0.68 ± 0.06 | 15.71, 2 | 0.555 | -0.04 ± 0.01 | -0.03 ± 0.02 | 0.35, 1 | linear | **<0.001** | -0.76 ± 0.06 | -0.81 ± 0.09 | 21.28, 2 | 0.038 | -0.04 ± 0.02 | 0.02 ± 0.02 | 4.29, 1 | linear | **0.019** | -1.06 ± 0.12 | -0.68 ± 0.07 | 7.97, 2 | **0.006** | 0.07 ± 0.03 | -0.03 ± 0.02 | 7.61, 1 |
|  | | | | | | | | | | | | | | | | | | | | | | | | | | | |
|  | **LU vs. LI** | | | | | | | | | **LU vs. MV** | | | | | | | | | **LI vs. MV** | | | | | | | | |
|  | **Group effect** | | | | | **Interaction with age** | | | | **Group effect** | | | | | **Interaction with age** | | | | **Group effect** | | | | | **Interaction with age** | | | |
|  | *Model order* | *p*-value | LI intercept  (± SD) | LU intercept  (± SD) | Log likelihood, df | *p*-value | LI  age slope (± SD) | LU age slope (± SD) | Log likelihood, df | *Model order* | *p*-value | LI intercept  (± SD) | LU intercept  (± SD) | Log likelihood, df | *p*-value | LI  age slope (± SD) | LU age slope (± SD) | Log likelihood, df | *Model order* | *p*-value | MV intercept  (± SD) | LI intercept  (± SD) | Log likelihood, df | *p*-value | MV  age slope (± SD) | LI  age slope (± SD) | Log likelihood, df |
| **Whole-brain delta power** | linear | 0.027 | 1.20 ± 0.05 | 1.22 ± 0.04 | 7.25, 2 | 0.193 | -0.01 ± 0.01 | -0.03 ± 0.01 | 1.69, 1 | linear | 0.027 | 1.20 ± 0.05 | 1.22 ± 0.04 | 7.25, 2 | 0.193 | -0.01 ± 0.01 | -0.03 ± 0.01 | 1.69, 1 | linear | 0.791 | 1.14 ± 0.09 | 1.20 ± 0.06 | 0.47, 2 | 0.494 | 0.00 ± 0.02 | -0.01 ± 0.02 | 0.47, 1 |
| **Whole-brain theta power** | linear | 0.118 | 0.83 ± 0.05 | 0.89 ± 0.04 | 4.27, 2 | 0.098 | -0.01 ± 0.01 | -0.03 ± 0.01 | 2.75, 1 | linear | 0.118 | 0.83 ± 0.05 | 0.89 ± 0.04 | 4.27, 2 | 0.098 | -0.01 ± 0.01 | -0.03 ± 0.01 | 2.75, 1 | linear | 0.879 | 0.87 ± 0.08 | 0.83 ± 0.06 | 0.26, 2 | 0.621 | -0.02 ± 0.02 | -0.01 ± 0.01 | 0.25, 1 |
| **Whole-brain alpha power** | linear | 0.221 | 0.23 ± 0.05 | 0.33 ± 0.04 | 3.02, 2 | 0.082 | 0.01 ± 0.01 | -0.02 ± 0.01 | 3.02, 1 | linear | 0.221 | 0.23 ± 0.05 | 0.33 ± 0.04 | 3.02, 2 | 0.082 | 0.01 ± 0.01 | -0.02 ± 0.01 | 3.02, 1 | linear | 0.974 | 0.25 ± 0.08 | 0.23 ± 0.06 | 0.05, 2 | 0.823 | 0.00 ± 0.02 | 0.01 ± 0.01 | 0.05, 1 |
| **Whole-brain beta power** | linear | 0.121 | -0.35 ± 0.07 | -0.24 ± 0.05 | 4.22, 2 | 0.077 | 0.01 ± 0.02 | -0.02 ± 0.01 | 3.12, 1 | linear | 0.121 | -0.35 ± 0.07 | -0.24 ± 0.05 | 4.22, 2 | 0.077 | 0.01 ± 0.02 | -0.02 ± 0.01 | 3.12, 1 | linear | 0.519 | -0.47 ± 0.10 | -0.35 ± 0.07 | 1.31, 2 | 0.262 | 0.04 ± 0.03 | 0.01 ± 0.02 | 1.26, 1 |
| **Whole-brain gamma power** | linear | 0.110 | -0.81 ± 0.09 | -0.68 ± 0.07 | 4.42, 2 | 0.110 | 0.02 ± 0.02 | -0.03 ± 0.02 | 2.55, 1 | linear | 0.110 | -0.81 ± 0.09 | -0.68 ± 0.07 | 4.42, 2 | 0.110 | 0.02 ± 0.02 | -0.03 ± 0.02 | 2.55, 1 | linear | 0.250 | -1.04 ± 0.12 | -0.81 ± 0.09 | 2.77, 2 | 0.225 | 0.07 ± 0.04 | 0.02 ± 0.02 | 1.47, 1 |

**Table S5**. Results of the mixed model analysis comparing resting-state EEG power between the TD and the three ASD language profiles, two by two. Note: P-value group effect evaluates the difference between power in the two groups whereas p-value interaction refers to the difference in the shape of the resting-state EEG power trajectory. The significance threshold was fixed at 0.05. P-values are adjusted for false discovery rate (FDR); bold values indicate statistically significant results. TD = Typical development; LU = Language Unimpaired; LI = Language Impaired; MV = Minimally-Verbal.

**Supplementary Figures**

**Figure S5.** Whole-brain resting-state EEG power spectra over the full frequency range. Power spectral density from 2-48 Hz for TD (typically developing, red), LU (language unimpaired, blue), LI (language impaired, green), and MV (minimally verbal, purple) groups. Lines represent group mean trajectories and shaded areas represent 95% confidence intervals. Power values are log10 transformed (μV2/Hz). The frequency range excludes the 49-51 Hz band due to notch filtering applied to remove electrical line noise.

**
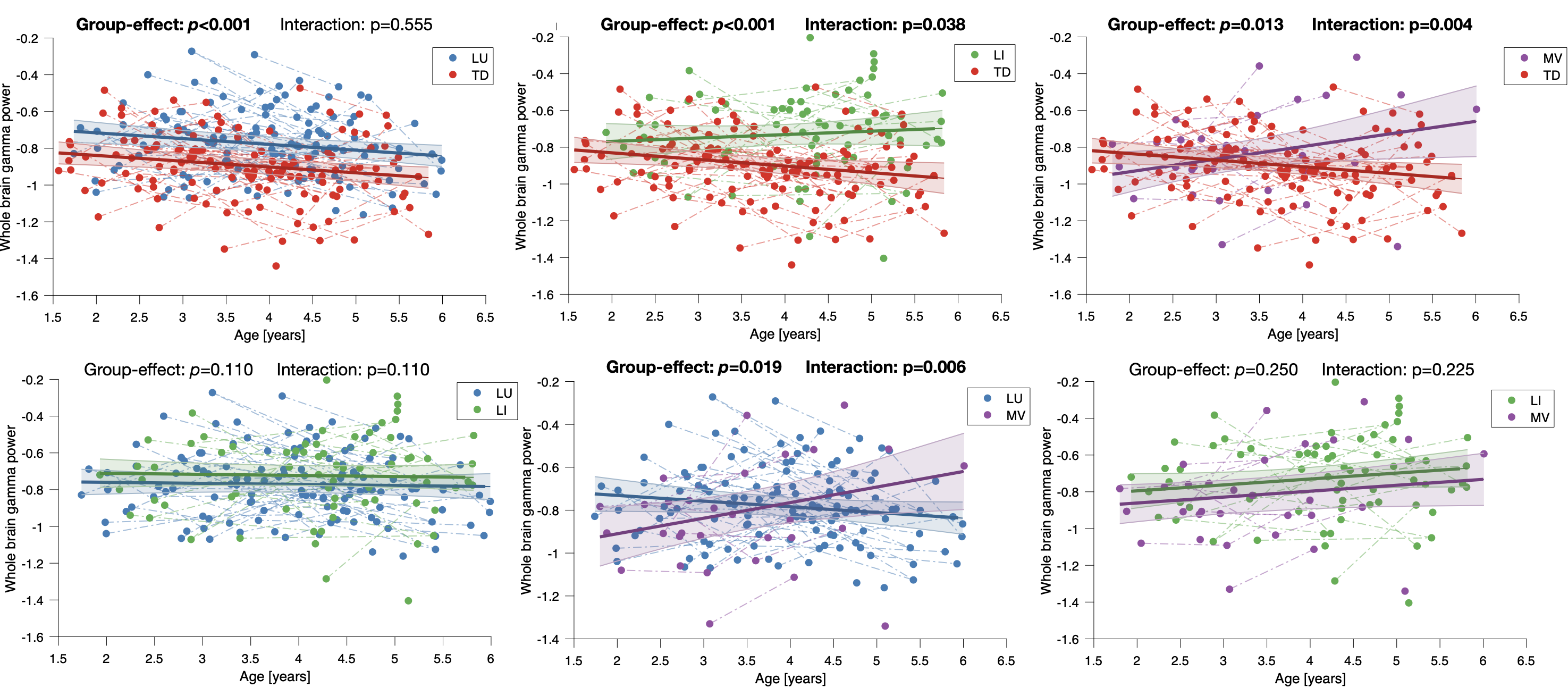
**

**Figure S6.** Two-by-two comparisons of whole-brain resting-state EEG gamma power (30-50Hz) of TD and of the three ASD language profiles (TD *vs.* LU; TD *vs*. LI; TD *vs*. MV; LU *vs.* LI; LU *vs.* MV; LI vs. MV).

The colored bands around the estimated group-level trajectory indicate the 95% confidence interval. TD: typical development; LU: language unimpaired; LI: language impaired; MV: minimally-verbal.

**
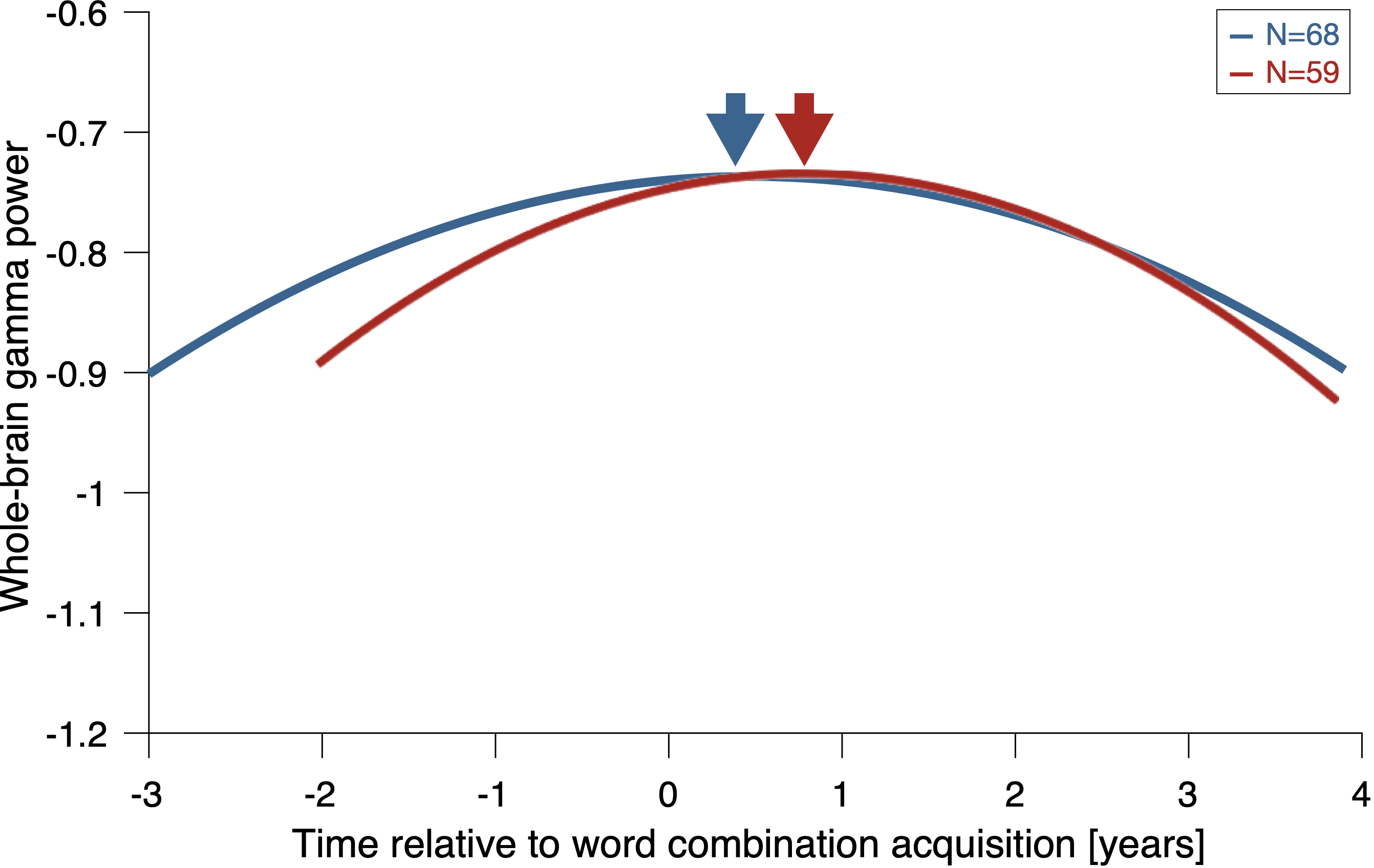
**

**Figure S7.** Whole-brain resting-state EEG gamma power trajectory in autistic children as a function of time relative to word combination acquisition (in years). The blue curve represents all autistic children with at least two consecutive time points (N = 68; 168 time points). The red curve represents the subset of children who acquired word combinations during the protocol (N = 59; 146 time points). The x-axis indicates time relative to individual word combination acquisition (in years).


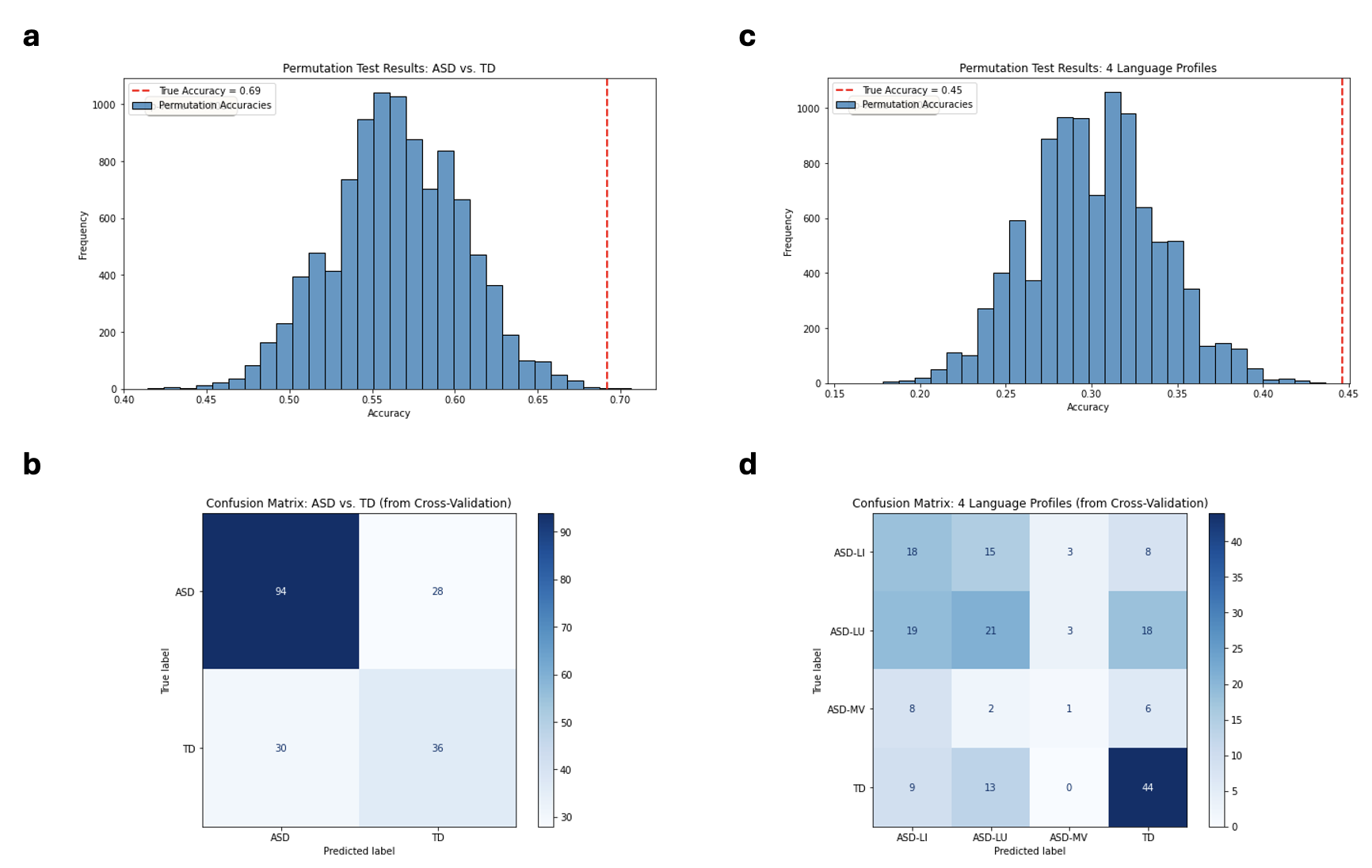


**Figure S8.** This figure displays the performance of the machine learning models for both the binary (ASD vs. TD) and multi-class (4 Language Profiles) classification tasks. Panels (a) and (c) show the results of the permutation tests used to assess statistical significance of the binary and 4-ways classification respectively. The histograms represent the null distribution of accuracies generated from 10,000 permutations with shuffled labels. The red dashed line in each plot indicates the true accuracy achieved by the model on the correctly labeled data. In both analyses, the true accuracy falls far outside the range of accuracies expected by chance, demonstrating the models' significant predictive power. Panels (b) and (d) present the confusion matrices, which provide a detailed summary of prediction performance from the out-of-sample cross-validation tests. (b) This matrix shows the model's ability to distinguish between individuals with Autism Spectrum Disorder (ASD) and Typically Developing (TD) controls. (d) This matrix illustrates the model's performance in the more challenging task of classifying individuals into one of three ASD language subgroups (LI: Language Impaired, LU: Language Unimpaired, MV: Minimally Verbal) or the TD group.
